# ITGA5 is a prognostic biomarker and correlated with immune infiltration in gastrointestinal tumors

**DOI:** 10.1186/s12885-021-07996-1

**Published:** 2021-03-12

**Authors:** Hai Zhu, Gang Wang, Haixing Zhu, Aman Xu

**Affiliations:** 1 Department of General Surgery, The First Affiliated Hospital of Anhui Medical University, Hefei, 230001 People’s Republic of China; 2 Department of General Surgery, The Fourth Affiliated Hospital of Anhui Medical University, Hefei, 230001 People’s Republic of China; 3 Department of Gastrointestinal Surgery, The First Affiliated Hospital of USTC, Division of Life Sciences and Medicine, University of Science and Technology of China, Hefei, 230031 People’s Republic of China

**Keywords:** ITGA5, Gastrointestinal tumor, Immune, Prognosis, Tumor-associated macrophages

## Abstract

**Background:**

Integrin Subunit Alpha 5 (ITGA5), belongs to the integrin alpha chain family, is vital for promoting cancer cell invasion, metastasis. However, the correlation between ITGA5 expression and immune infiltration in gastrointestinal tumors remain unclear.

**Methods:**

The expression level of ITGA5 was detected by Oncomine and Tumor Immune Estimation Resource (TIMER). The association between ITGA5 and prognosis of patients was identified by Kaplan–Meier plotter, Gene Expression Profiling Interactive Analysis 2 (GEPIA2) and PrognoScan. We evaluated the correlation between ITGA5 expression and immune infiltrating level via TIMER. Besides, TIMER, immunohistochemistry (IHC) staining and western blot were used to explore correlations between ITGA5 expression and markers of immune infiltrates cells. Furthermore, we constructed protein-protein interaction (PPI) network and performed functional enrichment by GeneMANIA and Metascape.

**Results:**

ITGA5 was generally overexpressed and correlated with worse prognosis in multiple types of gastrointestinal tumors. In addition, ITGA5 expression level was significantly associated with tumor purity and immune infiltration levels of different immune cells in gastrointestinal tumors. Interestingly, immune markers for monocytes, tumor - associated macrophages (TAMs), macrophages 2 (M2) cells and T-helper 2 (Th2) cells were found to be significantly and positively correlated with ITGA5 expression levels in colon and gastric cancer. Results from IHC staining and western blot further proved that markers of Th2 and M2 cell were significantly increased in gastric cancer patients with high ITGA5 expression levels. Lastly, interaction network and function enrichment analysis revealed ITGA5 was mainly involved in “integrin mediated signaling pathway”, “leukocyte migration”, “cell-substrate adhesion”.

**Conclutions:**

Our study demonstrated that ITGA5 may act as an essential regulator of tumor immune cell infiltration and a valuable prognostic biomarker in gastrointestinal tumors. Additional work is needed to fully elucidate the underlying mechanisms behind these observations.

**Supplementary Information:**

The online version contains supplementary material available at 10.1186/s12885-021-07996-1.

## Background

Gastrointestinal tumors are a common malignant tumor affecting individuals worldwide [1]. The high mortality rate associated with gastrointestinal tumors is a result of the tumor invading and metastasizing to other tissues. The increasing resistance observed in gastrointestinal tumors receiving chemotherapy and targeted therapy has led to an increased challenge when treating this disease [2–4]. There is extensive evidence that tumor-infiltrating lymphocytes, such as tumor - associated macrophages (TAMs), influence both the prognosis and efficacy of chemotherapy and immunotherapy [5, 6], as well as tumor angiogenesis, progression and metastasis [7]. Therefore, it is crucial to identify novel immune-related therapeutic targets and elucidate the potential mechanisms behind the immune interactions linked to gastrointestinal tumors.

Integrins are a large family of heterodimeric integral membrane proteins that function in cell surface adhesion and signaling pathways. Integrin Subunit Alpha 5 (ITGA5), primarily binds to Integrin Subunit Beta 1 (ITGB1) to form a α5β1 heterodimer [8, 9]. Recently, it has become clear that ITGA5 is essential for cancer proliferation, migration, invasion and metastasis [10–13]. In addition, ITGA5 was shown to maintain cancer cell stemness and chemotherapy resistance [14, 15]. Interestingly, ITGA5 was identified as a critical factor for promoting the differentiation of human mesenchymal stromal cells in vivo [16]. Recent studies revealed that ITGA5 plays a crucial role in different tumor cell subgroups and cell-cell interactions. Importantly, there is evidence revealing that immune components existing in the tumor microenvironment (TME) comprehensively regulate the biological behavior of the tumor through mutual interactions [17]. Furthermore, immunotherapy, especially the application of immune checkpoint modulators, is an efficient treatment strategy for solid tumors [18, 19].

However, the important role of ITGA5 in gastrointestinal tumors and its relationship with tumor immunity are still unclear. Here, we explored the expression level, prognostic value, protein interaction network and enrichment analysis of ITGA5 in gastrointestinal tumors. More importantly, the correlation between ITGA5 and tumor-infiltrating immune cells and markers in different tumor microenvironments was also analyzed. Our result supported the pro-oncogenic effect of ITGA5 and revealed the overexpression of ITGA5 may have a potential and important relationship with tumor-immune infiltration.

## Methods

### Clinical tissue specimens

The tissue samples used for immunohistochemistry (IHC) staining and western blot were collected from patients diagnosed with gastric cancer at the First Affiliated Hospital of Anhui Medical University. All patients have already provided written informed consent. This work was approved by the Academic Committee of The First Affiliated Hospital of Anhui Medical University (certification no. Quick-PJ 2020-11-14) and was conducted following the Declaration of Helsinki.

### Oncomine analysis

Expression of ITGA5 mRNA levels were analyzed to compare normal and tumor tissues in various cancer types using the Oncomine database (https://www.oncomine.org) [20, 21]. The filter condition was set to a *P* value < 0.001, fold change > 1.5, gene rank: 10%, data type: mRNA.

### PrognoScan analysis

PrognoScan database (http://www.abren.net/PrognoScan) was used to identify the correlation between ITGA5 expression and prognosis in various cancers across a large collection of publicly available microarray datasets [22]. The threshold was adjusted to a Cox *P*-value < 0.05.

### Kaplan-Meier plotter analysis

Kaplan-Meier plotter (https://kmplot.com) possesses gene expression data and survival information associated with 1065 gastric cancer patients [23]. To evaluate differential expression of ITGA5 mRNA levels and the effects on patient progression, Kaplan–Meier survival curves for overall survival (OS) and disease or progression free survival (DFS or PFS) were generated for patients containing low and high ITGA5 mRNA expression levels. In addition, we used the Kaplan-Meier Plotter to evaluate the relationship between the expression of ITGA5 and the clinicopathological characteristics of gastric cancer patients. Results contained a hazard ratio (HR) with a 95% confidence interval (CI) and log rank *P* value.

### TIMER analysis

Tumor Immune Estimation Resource (TIMER) database (https://cistrome.shinyapps.io/timer) is a publicly available comprehensive resource for systematic analysis of tumor immune-infiltrates [24]. It includes 10,897 samples across 32 different cancer types from The Cancer Genome Atlas (TCGA). The correlation between ITGA5 expression levels and immune infiltrates including B cells, CD4+ T cells, CD8+ T cells, neutrophils, macrophages and dendritic cells, were explored using the gene module in various cancer types. The correlation between genes markers of tumor-infiltrating immune cells and ITGA5 expression was analyzed using the correlation module. Gene markers included CD8+ T cells, general T cells, B cells, monocytes, TAMs, M1 macrophages, M2 macrophages, neutrophils, natural killer (NK) cells, dendritic cells (DCs), T-helper 1 (Th1) cells, T-helper 2 (Th2) cells, follicular helper T (Tfh) cells, T-helper 17 (Th17) cells, Tregs and exhausted T cells. ITGA5 expression was plotted on the X-axis and the expression of related marker genes were represented as gene symbols on the Y-axis. Correlation coefficients were estimated using Spearman’s correlation method. Gene expression levels were shown as log2 RSEM.

### GEPIA2 analysis

Gene expression Profiling Interactive Analysis 2 (GEPIA2) (http://gepia2.cancer-pku.cn) is an updated and enhanced version of GEPIA that supports 198,619 isoforms and 84 cancer subtypes [25, 26]. In our study, the “Expression DIY” and “Survival” module of GEPIA2 were used to determine the correlation between ITGA5 expression and prognosis in pancreatic and esophageal cancers.

### Immunohistochemistry staining analysis

Collected tissue specimens were formalin fixed and embedded with paraffin. Tissue sections (4 μm thickness) were used in IHC staining analyses as previously described [27]. The intensity of immunostaining and the percentage of positive cells were used to determine the immunoreactivity to proteins. The density grading of intensity was analyzed as follows: 0:no staining, 1:weak staining; 2: medium staining, and 3: strong staining. The proportion of positive cells was further classified as 0: < 1%; 1: 1–30%, 2: 30–70%, and 3:> 70%. Semi-quantitative assessment and final immunostaining scores were determined by multiplying the proportion score and intensity scores. Scores 0, 1, 2 and 3 indicated low expression levels, while scores 4, 6 and 9 indicated high expression levels. IHC results were evaluated by two pathologists using a blind test and differences were resolved through consensus. Antibody information is listed in Additional file: Table S1.

### Western blot analysis

All the collected tissues were cut and ground into a homogenate. Using RIPA buffer (Radio-Immunoprecipitation assay buffer, Beyotime) to lyse aggregated proteins in the cells. In addition, the concentrations were detected with the help of the BCA Protein Assay Kit (Beyotime). Segregation of equivalent quantities of proteins was made using the SDS-PAGE, followed by transferring to PVDF membranes (Millipore, Boston, MA, USA). After that, the membrane was incubated with the targeted primary antibody overnight at 4 °C. After washing, incubation of the membranes was performed with the corresponding secondary antibody for 2 h at the room temperature. GAPDH was used as an internal loading control. The findings were analyzed with the help of the ECL detection system (Pierce Biotech, Rockford, IL, USA). Antibody information is listed in Additional file: Table S2.

### GeneMANIA analysis

GeneMANIA (http://genemania.org/) is used to construct a protein-protein interaction (PPI) network and analyze the function of interactive genes [28]. This online tool uses bioinformatic methods to display a list of interacting genes, including gene co-expression, physical interactions, gene co-localization, gene enrichment analysis and website prediction. GeneMANIA was used to construct the PPI network for ITGA5.

### Metascape analysis

Metascape (https://metascape.org/gp/index.html) is a gene function annotation website with obvious advantages of wide coverage and fast updating [29]. It integrates multiple authoritative data resources such as Gene Ontology (GO), Kyoto Encyclopedia of Genes and Genomes (KEGG) pathway, UniProt and DrugBank to complete thorough pathway enrichment and biological process annotation. Genes interacting with ITGA5 were put into Metascape to perform GO and KEGG annotation.

### Statistical analysis

All statistical analyses were performed using SPSS 21.0 software (IBM Corp; Armonk, NY). Survival curves and relative results generated from PrognoScan, Kaplan-Meier Plotter and GEPIA2 databases were shown with HR and *P* or Cox *P* values from a log-rank test. Correlation analyses in TIMER were evaluated using Spearman’s correlation. Associations between ITGA5 expression and IHC results in patients were evaluated using Pearson’s χ2 test. Student’s t-test was used to determine the significance for the western blot experiments. *P*-value of < 0.05 was considered as statistically significant.

## Results

### Pan-cancer analysis of ITGA5 expression levels

**Fig. 1:**
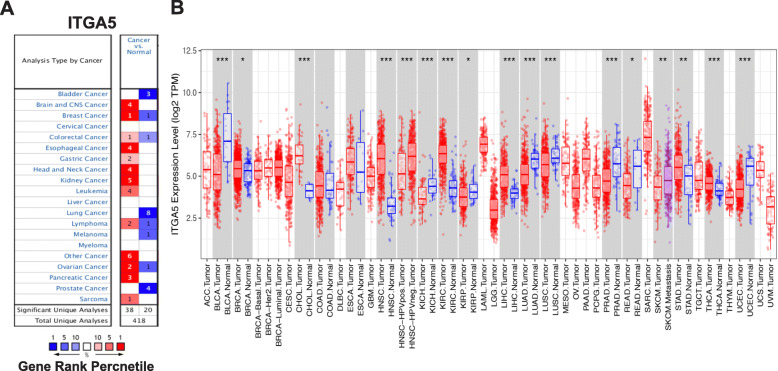
ITGA5 expression in different types of human cancers. **a** Different expression of ITGA5 between tumors and normal tissues in Oncomine database. The filter condition was set as: *p* value < 0.0001, fold change > 2, gene rank: 10%, data type: mRNA. **b** Different expression of ITGA5 between tumors and normal tissues in TIMER database (*P < 0.05, **P < 0.01, ***P < 0.001)

To determine ITGA5 mRNA expression levels in both normal and tumor tissues, Oncomine was interrogated to analyze ITGA5 expression in various cancer types. The expression levels of ITAG5 were greater in colorectal, esophageal, gastric and pancreatic cancers relative to normal tissues (Fig. 1a). In addition, the transcriptional levels for ITGA5 were analyzed using RNA-seq data for multiple malignancies in the TCGA with the use of TIMER. Results revealed significant differences in ITGA5 expression levels when comparing normal and tumor tissues. Hepatocellular carcinoma (LIHC) and gastric adenocarcinoma (STAD) were significantly higher compared with normal tissue levels (Fig. 1b).

### Correlation between ITGA5 expression levels and patient prognosis

**Fig. 2:**
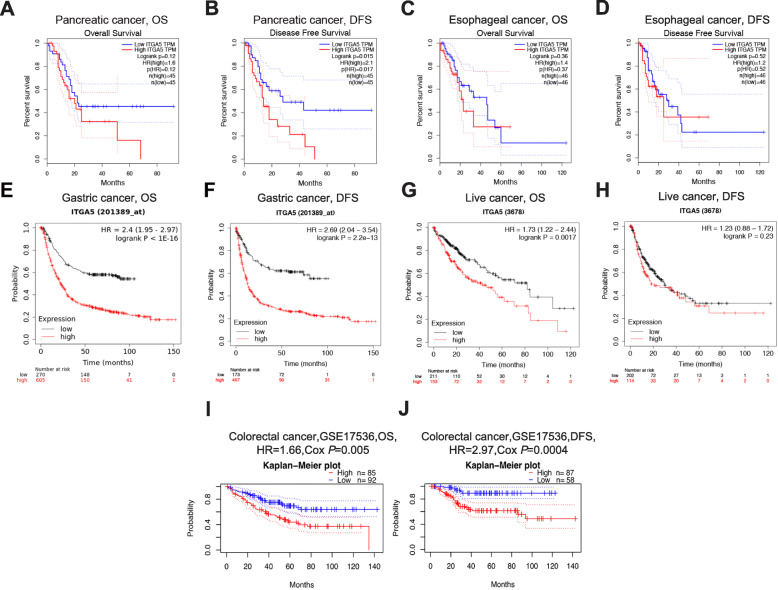
The prognostic value of ITGA5 expression in gastrointestinal cancers. **a-d** Correlation between ITGA5 expression and prognosis of pancreatic cancer and esophageal cancer in GEPIA2; **e-h** Correlation between ITGA5 expression and prognosis of gastric cancer and liver cancer in Kaplan-Meier Plotter; **i-j** Correlation between ITGA5 expression and prognosis of colorectal cancer in PrognoScan. OS, overall survival; DFS, disease free survival; HR: Hazard ratio

To further identify the prognostic potential of ITGA5 in various cancers, clinical data from TCGA were used to explore the prognostic value of ITGA5 expression level using GEPIA2. High expression of ITGA5 was marginally correlated with poor prognosis in pancreatic cancer (OS HR = 1.6, *P* = 0.12; DFS HR = 2.1, *P* = 0.015) (Fig. 2a-b) and esophageal cancer (OS HR = 1.4, *P* = 0.37; DFS HR = 1.2, *P* = 0.52) (Fig. 2c-d). Subsequently, the Kaplan-Meier plotter database was used to determine the prognostic value of ITGA5. Significant differences were observed between ITGA5 expression and prognosis in gastric cancer (OS HR = 2.4, 95% CI = 1.96 to 2.97, *P* < 1e-16; DFS HR = 2.69, 95% CI = 2.04 to 3.54, *P* = 2.2e-13) (Fig. 2e-f) and live cancer (OS HR = 1.73, 95% CI = 1.22 to 2.44, *P* = 0.0017; DFS HR = 1.23, 95% CI = 0.88 to 1.72, *P* = 0.23) patients (Fig. 2g-h). In addition, the PrognoScan database was used to investigates the relationship between ITGA5 mRNA levels and the survival of cancer patients using high-throughput analysis and detailed clinical prognosis data. ITGA5 expression was found to significantly impact prognosis in colorectal. Specifically, the cohorts (GSE17536) [30] included 177 samples at different stages of colorectal cancer and showed a remarkable association between high ITGA5 expression levels and poor prognosis (OS HR = 1.66, 95% CI = 1.16 to 2.37, Cox *P* = 0.005; DFS HR = 2.97, 95% CI = 1.70 to 5.21, Cox *P* = 0.0004) (Figs. 2i–j) in colorectal cancer.

Interestingly, we also found that ITGA5 also has a significant impact on the prognosis of patients in some non-gastrointestinal tumors. High expression of ITGA5 was also associated with poor prognosis in lung cancer (OS HR = 1.6, 95% CI = 1.4 to 1.81, *P* = 5.9e-13; DFS HR = 1.75, 95% CI = 1.44 to 2.12, *P* = 1.2e-08) and breast cancer (OS HR = 1.21, 95% CI = 0.96 to 1.52, *P* = 0.11; DFS HR = 1.28, 95% CI = 1.14 to 1.44, *P* = 4.1e-05) (Additional file: Fig. S1). Similarly, the cohorts (GSE26712) [31] showed that high ITGA5 expression levels were correlated with poor prognosis (OS HR = 1.58, 95% CI = 1.06 to 2.36, Cox *P* = 0.025; DFS HR = 1.54, 95% CI = 1.06 to 2.24, Cox *P* = 0.025) in ovarian cancers (Additional file: Fig. S1).

### Relationship between ITGA5 expression and immune infiltration levels in gastrointestinal cancers

**Fig. 3:**
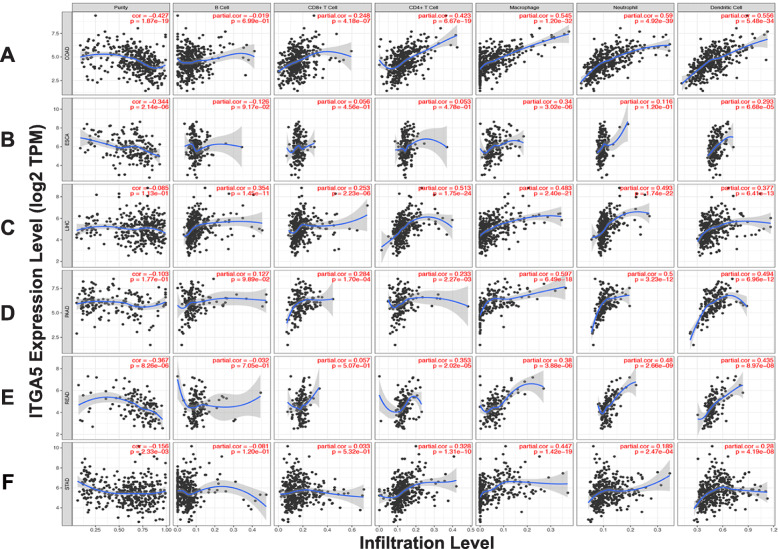
Correlation of ITGA5 expression with immune infiltration level in Gastrointestinal tumors. **a** Colon adenocarcinoma (COAD); **b** Esophageal carcinoma (ESCA); **c** Liver hepatocellular carcinoma (LIHC); **d** Pancreatic adenocarcinoma (PAAD); **e** Rectum adenocarcinoma (READ); **f** Stomach adenocarcinoma (STAD)

Recent work has shown that gastrointestinal tumors exhibit extensive immune infiltration characteristics [32–34]. Therefore, to better understand the role of ITGA5 in gastrointestinal tumors, the relationship between ITGA5 expression and immune infiltration in gastrointestinal tumors was investigated using the TIMER database. ITGA5 expression was found to be significantly correlated with tumor purity in colon adenocarcinoma (COAD), esophageal carcinoma (ESCA), STAD and rectum adenocarcinoma (READ) (*P* < 0.05), CD8+ cell in COAD, LIHC and pancreatic adenocarcinoma (PAAD), CD4 + T cells in COAD, PAAD, LIHC, READ and STAD (*P* < 0.05) (Fig. 3a-f). Furthermore, ITGA5 expression was significantly associated with macrophages and dendritic cells in COAD, ESCA, READ, LIHC, PAAD and STAD (*P* < 0.05) (Fig. 3a-f). ITGA5 expression was also significantly associated with neutrophils in COAD, LIHC, READ, PAAD and STAD (*P* < 0.05) (Fig. 3a-f).

Specifically, ITGA5 expression levels showed significantly positive correlations with infiltrating levels of CD8 + T cells (r = 0.248, *P* = 4.18e-07), CD4+ T cells (r = 0.423, *P* = 6.67e-19), macrophages (r = 0.545, *P* = 1.20e-32), neutrophils (r = 0.590, *P* = 4.92e-39) and dendritic cells (r = 0.556, *P* = 5.48e-34) in COAD (Fig. 3a). Similarly, positive correlations were identified with infiltrating levels of CD4+ T cells (r = 0.426, *P* = 1.52e-17), macrophages (r = 0.447, *P* = 1.42e-19), neutrophils (r = 0.189, *P* = 2.47e-04) and dendritic cells (r = 0.28, *P* = 4.19e-08) in STAD (Fig. 3f). These results revealed that ITGA5 expression is closely associated with immune infiltration in gastrointestinal tumors.

### Correlation between ITGA5 expression levels and immune markers

**Fig. 4:**
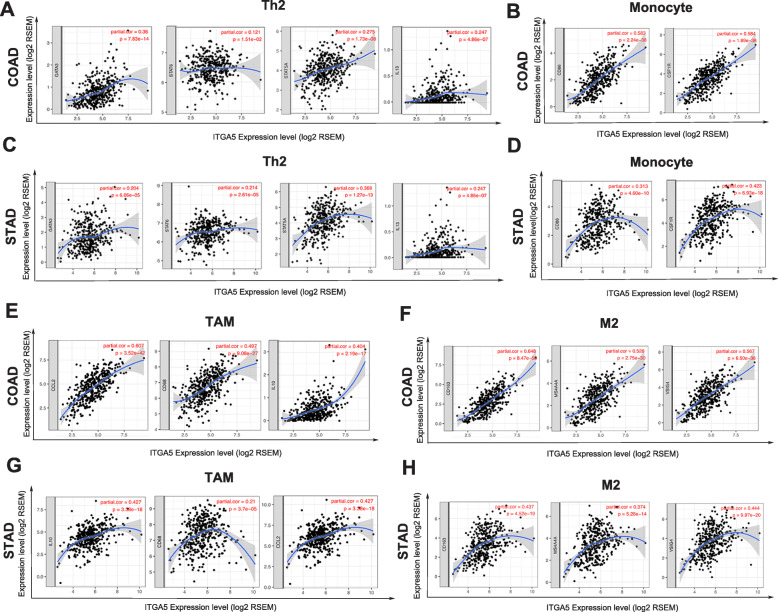
Correlation analysis between ITGA5 expression and immune marker set in STAD and COAD. **a** Scatterplots of correlations between ITGA5 expression and gene markers of Th2 cells in COAD; **b** Scatterplots of correlations between ITGA5 expression and gene markers of Monocyte in COAD; **c** Scatterplots of correlations between ITGA5 expression and gene markers of Th2 cells in STAD; **d** Scatterplots of correlations between ITGA5 expression and gene markers of Monocyte in STAD; **e** Scatterplots of correlations between ITGA5 expression and gene markers of TAM in COAD; **f** Scatterplots of correlations between ITGA5 expression and gene markers of M2 in COAD; **g** Scatterplots of correlations between ITGA5 expression and gene markers of TAM in STAD; **h** Scatterplots of correlations between ITGA5 expression and gene markers of M2 cells in STAD

Gastric cancer and colorectal cancer are the most common gastrointestinal-related malignancies. Therefore, COAD and STAD were chosen to further investigate the relationship between ITGA5 expression and immune marker genes of diverse immune cells using TIMER databases. The immune cells analyzed included CD8+ T cells, general T cells, different functional T cells, B cells, monocytes, TAMs, M1 macrophages and M2 macrophages, neutrophils, NK cells and dendritic cells. After adjusting correlation by purity, ITGA5 expression was found to be significantly correlated with immune markers of most immune cells in STAD and COAD (Table 1 and Fig. 4a-h). Immune markers for monocytes, TAMs, M2 and Th2 phenotypes were found to be significantly and positively correlated with ITGA5 expression levels in COAD and STAD (*P* < 0.05; Figs. 4a-h).

**Table 1:** Correlation analysis between ITGA5 and markers of immune cells in TIMER database

Description	Gene markers	COAD				STAD			
		None		Purity		None		Purity	
		Cor	***P***	Cor	***P***	Cor	***P***	Cor	***P***
CD8+ T cell	CD8A	0.34	***	0.235	***	0.152	**	0.134	**
	CD8B	0.22	***	0.168	***	0.094	0.055	0.094	0.066
T cell (general)	CD3D	0.284	***	0.127	*	0.103	*	0.07	0.175
	CD3E	0.381	***	0.241	***	0.381	***	0.102	0.046
	CD2	0.348	***	0.216	***	0.348	***	0.118	0.021
B cell	CD19	0.226	***	0.072	0.150	0.226	***	0.205	***
	CD79A	0.303	***	0.147	**	0.303	***	0.146	**
Monocyte	CD86	0.651	***	0.583	***	0.651	***	0.313	***
	CD115 (CSF1R)	0.65	***	0.584	***	0.65	***	0.423	***
TAM	CCL2	0.607	***	0.607	***	0.659	***	0.427	***
	CD68	0.556	***	0.497	***	0.556	***	0.21	***
	IL10	0.461	***	0.404	***	0.392	***	0.388	***
M1 Macrophage	INOS (NOS2)	0.064	0.172	−0.125	*	0.035	0.475	0.042	0.411
	IRF5	0.296	***	0.323	***	0.193	***	0.197	***
	COX2(PTGS2)	0.41	***	0.359	***	0.402	***	0.398	***
M2 Macrophage	CD163	0.700	***	0.648	***	0.444	***	0.437	***
	VSIG4	0.633	***	0.567	***	0.437	***	0.444	***
	MS4A4A	0.597	***	0.526	***	0.378	***	0.374	***
Neutrophils	CD66b (CEACAM8)	−0.218	***	0.207	***	0.018	0.714	0.045	0.384
	CD11b (ITGAM)	0.684	***	0.624	***	0.427	***	0.418	***
	CCR7	0.405	***	0.274	***	0.319	***	0.292	***
Natural killer cell	KIR2DL1	0.224	***	0.173	***	0.11	*	0.117	****
	KIR2DL3	0.168	***	0.121	0.015	0.039	0.433	0.031	0.553
	KIR2DL4	0.222	***	0.135	**	−0.03	0.549	−0.046	0.375
	KIR3DL1	0.267	***	0.201	***	0.092	0.062	0.076	0.138
	KIR3DL2	0.249	***	0.177	0.177	0.08	0.102	0.074	0.149
	KIR3DL3	0.052	0.265	0.039	0.431	−0.069	0.159	−0.033	0.525
	KIR2DS4	0.194	***	0.162	**	0.051	0.296	0.055	0.284
Dendritic cell	HLA-DPB1	0.501	***	0.389	***	0.136	**	0.101	0.051
	HLA-DQB1	0.297	***	0.183	***	0.068	0.169	0.04	0.441
	HLA-DRA	0.46	***	0.355	***	0.076	0.120	0.044	0.394
	HLA-DPA1	0.486	***	0.385	***	0.09	0.069	0.056	0.281
	BDCA-1(CD1C)	0.304	***	0.199	***	0.246	***	0.213	***
	BDCA-4(NRP1)	0.83	***	0.794	***	0.639	***	0.63	***
	CD11c (ITGAX)	0.738	***	0.675	***	0.408	***	0.392	***
Th1	T-bet (TBX21)	0.43	***	0.336	***	0.183	***	0.172	***
	STAT4	0.343	***	0.231	***	0.222	***	0.222	***
	STAT1	0.451	***	0.411	***	0.076	0.124	0.071	0.169
	IFN-γ (IFNG)	0.242	***	0.183	***	−0.043	0.380	−0.052	0.312
	TNF-α (TNF)	0.383	***	0.334	***	0.236	***	0.204	***
Th2	GATA3	0.442	***	0.36	***	0.22	***	0.204	***
	STAT6	0.132	**	0.121	*	0.199	***	0.214	***
	STAT5A	0.307	***	0.275	***	0.367	***	0.368	***
	IL13	0.3	***	0.247	***	0.167	***	0.19	***
Tfh	BCL6	0.643	***	0.583	***	0.503	***	0.471	***
	IL21	0.202	***	0.154	**	0.029	0.563	0.012	0.809
Th17	STAT3	0.417	***	0.37	***	0.462	***	0.457	***
	IL17A	0.198	***	0.224	***	−0.103	*	−0.112	*
Treg	FOXP3	0.537	***	0.454	***	− 0132	0.244	−0.107	0.395
	CCR8	0.543	***	0.481	***	0.123	0.280	0.055	0.642
	STAT5B	0.356	***	0.385	***	0.381	***	0.418	***
	TGFβ (TGFB1)	0.69	***	0.606	***	0.6	***	0.591	***
T cell exhaustion	PD-1 (PDCD1)	0.395	***	0.28	***	0.175	***	0.17	***
	CTLA4	0.465	***	0.28	***	0.155	**	0.144	**
	LAG3	0.419	***	0.317	***	0.134	**	0.126	*
	TIM-3 (HAVCR2)	0.645	***	0.584	***	0.33	***	0.33	***
	GZMB	0.099	*	0.077	0.122	0.068	0.169	0.05	0.332

*COAD* Colon adenocarcinoma, *STAD* Stomach adenocarcinoma, None: age and tumor purity are not considered (it means that the tumor purity and age are not used to correct the results by using the partial Sperarman’s correlation when performing this association analysis); Purity: tumor purity is considered (it means the tumor purity is used to correct the results when performing correlation analysis); Cor: Correlation coefficient; *P*:*P* value; *P < 0.05, **P < 0.01, ***P < 0.001

### High ITGA5 expression affects the prognosis of gastric cancer patients exhibiting lymph node metastasis

To better understand the relationship between ITGA5 expression levels and clinicopathological features, further research was focused on gastric cancer. According to Kaplan-Meier Plotter database analyses, overexpression of ITGA5 is strongly correlated with the deterioration of OS and PFS based on gender, differentiation and Lauren classification (*P* < 0.05) (Table 2). In addition, stage I through IV cancer patients with high expression levels of ITGA5 showed worse OS (*P* < 0.05) (Table 2). Similarly, high expression levels of ITGA5 were associated with worse PFS in stage II through IV gastric cancer patients (*P* < 0.05) but not in stage I gastric cancer patients (Table 2). However, there was no strong correlation observed between ITGA5 expression levels and patient prognosis in stage N0 (OS HR = 1.92, *P* = 0.13; PFS HR = 1.95, *P* = 0.11), mixed Lauren classification (OS HR = 1.88, *P* = 0.22; PFS HR = 0.48, *P* = 0.16) or poor differentiation (OS HR = 0.75, *P* = 0.16; PFS HR = 1.39, *P* = 0.25) (Table 2). Moreover, high ITGA5 levels express the highest HR value of N1 with OS and PFS among four N categories (Table 2). These results indicate that the expression levels of ITGA5 are related to lymph node metastasis in gastric cancer patients.

**Table 2:** Correlation of ITGA5 and clinicopathological factors in STAD from Kaplan-Meier Plotter database

Clinicopathological characteristics	Overall survival (***n*** = 875)			Progression free survival (***n*** = 640)		
	N	Hazard ratio	***P***-value	N	Hazard ratio	***P***-value
**Gender**						
Female	236	2.74 (1.68–4.46)	2.6e-05	201	2.69 (1.58–4.58)	0.00014
Male	544	2.75 (2.12–3.57)	2.2e-15	437	2.55 (2.00–3.25)	4.2e-15
**Stage**						
1	67	3.62 (1.00–13.12)	0.036	60	2.42 (0.63–9.31)	0.19
2	140	2.18 (1.09–4.33)	0.023	131	1.96 (0.98–3.90)	0.051
3	305	2.31 (1.61–3.30)	2.4e-06	186	1.87 (1.29–2.72)	8e-04
4	148	1.78 (1.2–2.64)	0.0039	141	1.60 (1.07–2.38)	0.02
**Stage T**						
2	241	2.00 (1.29–3.11)	0.0015	239	1.98 (1.26–3.11)	0..0024
3	204	1.73 (1.19–2.51)	0.0036	204	1.37 (0.98–1.91)	0.065
4	38	2.73 (1.10–6.75)	0.025	39	3.39 (1.37–8.40)	0.0054
**Stage N**						
0	74	1.92 (0.82–4.46)	0.13	72	1.95 (0.85–4.49)	0.11
1	225	3.13 (1.99–4.94)	2.3e-07	222	2.84 (1.83–4.42)	1.2e-06
2	121	2.56 (1.62–4.05)	3.1e-05	125	2.08 (1.33–3.25)	0.00098
3	76	2.1 (1.22–3.61)	0.0061	76	1.84 (1.02–3.33)	0.04
1 + 2 + 3	422	2.14 (1.64–2.80)	9.8e-09	423	2.30 (1.66–3.20)	2.8e-07
**Stage M**						
0	444	2.31 (1.66–3.21)	2.6e-07	443	2.12 (1.55–2.91)	1.6e-06
1	56	2.66 (1.45–4.88)	0.0011	56	1.69 (0.92–3.12)	0.088
**Lauren classification**						
Intestinal	320	2.84 (2.07–3.90)	1.6e-11	263	2.70 (1.74–4.20)	4.2e-06
Diffuse	241	2.00 (1.33–2.99)	0.00061	231	2.10 (1.33–3.32)	0.0012
Mixed	32	1.88 (0.68–5.20)	0.22	28	0.48 (0.17–1.35)	0.16
**Differentiation**						
Poor	165	0.75 (0.50–1.12)	0.16	121	1.39 (0.79–2.45)	0.25
Moderate	67	2.16 (1.08–4.30)	0.025	67	2.23 (1.15–4.32)	0.015

*STAD* Stomach adenocarcinoma, *N* the number of patients

(Please put Table 2 at the end of the paragraph which begin with the subtitle “High ITGA5 expression affects the prognosis of gastric cancer patients exhibiting lymph node metastasis”)

### ITGA5 protein expression and M2 and Th2 immune marker genes in STAD

**Fig. 5:**
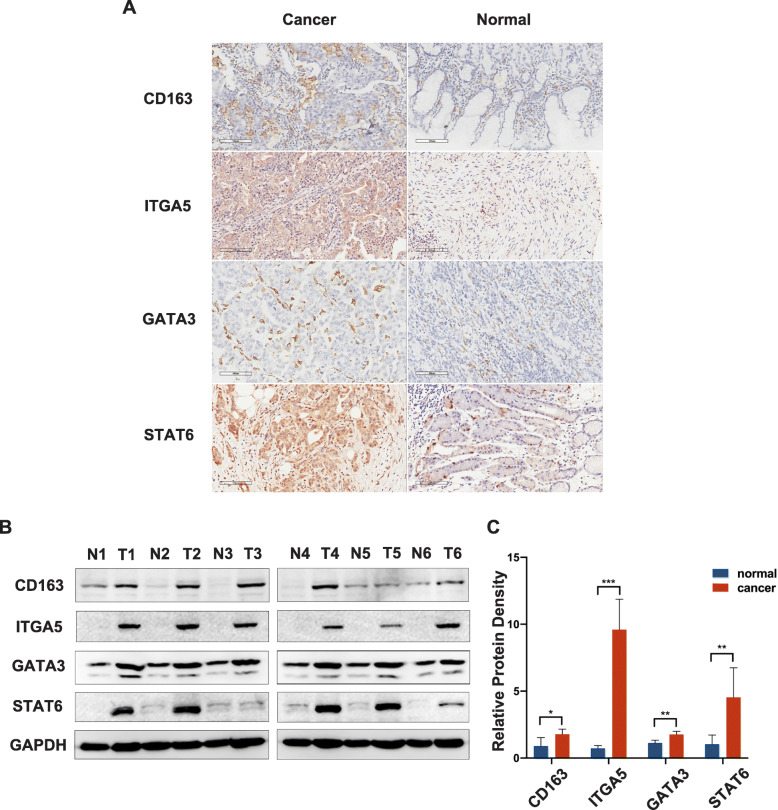
IHC and western blot analysis between ITGA5 and different markers of Th2 and M2 cells in gastric cancer tissues and adjacent normal tissues. **a** Representative IHC images of CD163, ITGA5, GATA3 and STAT6 in gastric cancer tissues and adjacent normal tissues; **b** Representative western blot images of CD163, ITGA5, GATA3 and STAT6 in 6 paired gastric cancer tissues (T) and adjacent normal tissues (N) (The full-length, original blots are presented in Additional file: Fig. S2); **c** Relative protein density of CD163, ITGA5, GATA3 and STAT6 in 6 paired gastric cancer tissues and adjacent normal tissues

To confirm the results obtained from publicly available databases, IHC were performed on adjacent normal and STAD tumor tissues obtained from 40 gastric patients. Compared with adjacent normal tissues, ITGA5, CD163, STAT6 and GATA3 levels were greater in most tumor tissues (Fig. 5a). In addition, these results revealed that the expression levels of CD163 (χ^2^ = 8.750, *P* = 0.003), STAT6 (χ^2^ = 8.174, *P* = 0.004) and GATA3 (χ^2^ = 5.079, *P* = 0.024) in the ITGA5 high-expression group were significantly higher than what was observed in the ITGA5 low-expression group (Table 3). Among the patients with higher ITGA5 expression levels, 12 patients showed higher CD163, STAT6 and GATA3 levels at the same time. In addition, we attempted to detect the proteins levels of ITGA5, CD163, STAT6 and GATA3 in patient tissues by western blot. As Fig. 5b, c shows, the expression levels in tumor tissues were indeed higher than that in adjacent normal tissues.

**Table 3:** The protein expression level of ITGA5 and immune marker genes of M2 and Th2 cells in STAD

		ITGA5 high (***n*** = 28)	ITGA5 low (***n*** = 12)	χ2	***P***-value
**CD163**	High(*n* = 24)	21	3	8.750	0.003
	Low (*n* = 16)	7	9		
**STAT6**	High(*n* = 29)	24	5	8.174	0.004
	Low (*n* = 11)	4	7		
**GATA3**	High(*n* = 24)	20	4	5.079	0.024
	Low(*n* = 16)	8	8		

(Please put Table 3 at the end of the paragraph which begin with the subtitle “ITGA5 protein expression and M2 and Th2 immune marker genes in STAD”)

### ITGA5 PPI network and enrichment analyses

**Fig. 6:**
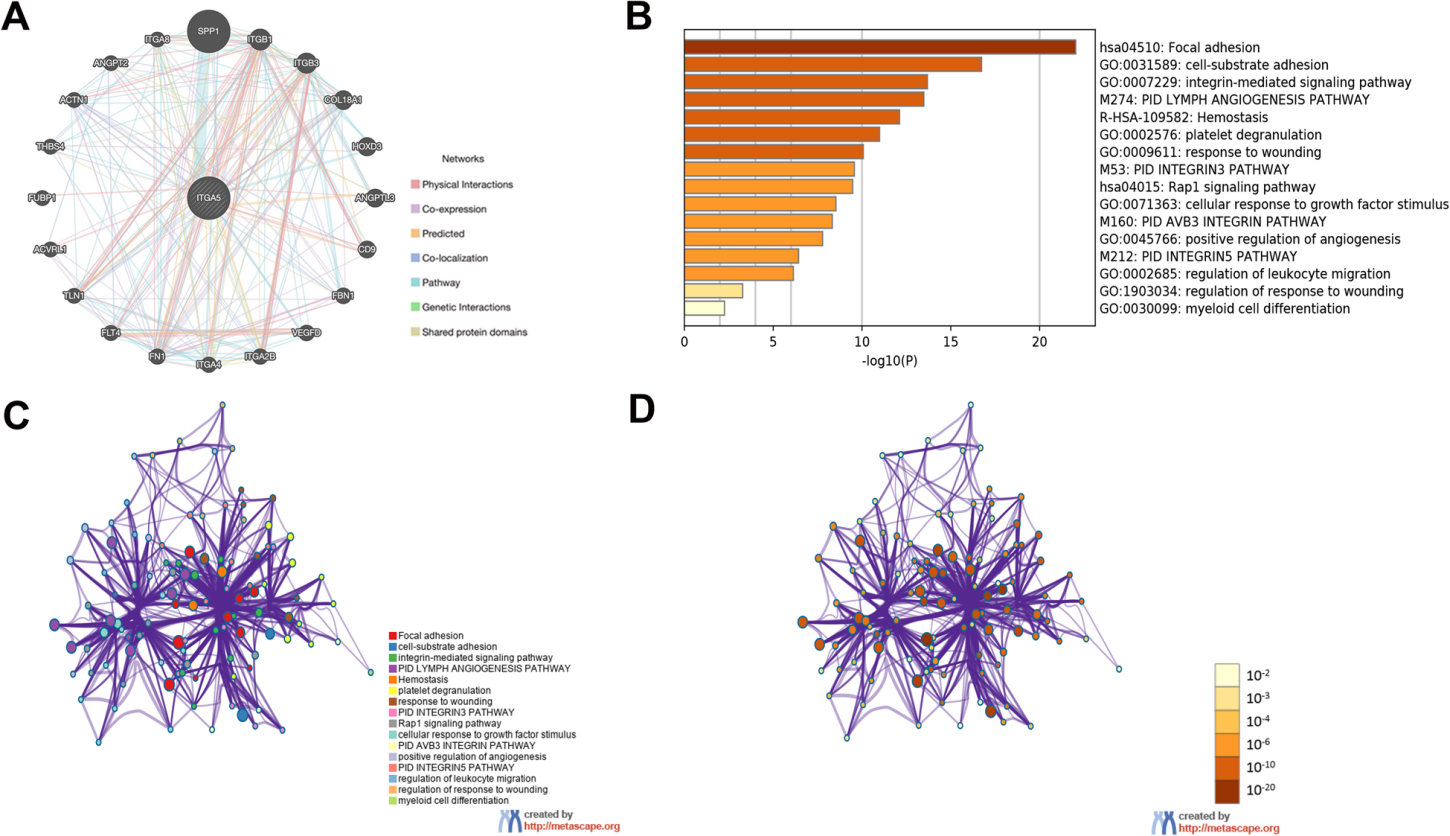
PPI network of ITGA5 and functional enrichment analysis. **a** PPI network of ITGA5 in GeneMANIA, different colors of the network edge indicate the bioinformatics methods applied: physical interactions, co-expression, predicted, co-localization, pathway, genetic interactions, and shared protein domains; **b** A heat map of GO and KEGG analysis of ITGA5 and it’s 20 most interactive genes, orange represents the enrichment terms colored by *p* values; **c,d** Interactive network of the top enrichment terms colored by cluster ID, different colors represent various enrichment pathways of ITGA5 correlated genes

PPI network analysis revealed interactions between ITGA5 and specific genes. As shown by GeneMANIA, genes interacting with ITGA5 included SPP1, ITGB1, ITGB3, COL18A1, HOXD3, ANGPTL3, CD9, FBN1, VEGFD, ITGA2B, ITGA4, FN1, FLT4, TLN1, ACVRL1, FUBP1, THBS4, ACTN1, ANGPT2 and ITGA8 (Fig. 6a).

To further predict enriched function and pathway information of ITGA5 interacting genes, GO function and KEGG pathway analyses were performed using Metascape. According to GO term analysis, genes interacting with ITGA5 in the PPI network are mainly related with cell-substrate adhesion, regulation of leukocyte migration, response to wounding and myeloid cell differentiation. KEGG pathway analysis showed these genes are enriched in focal adhesion, PID avb3 integrin pathway and PID integrin5 pathway. Based on these results, ITGA5 and its interacting proteins were shown to play an important role in the integrin mediated signaling pathway, leukocyte migration, cell-substrate adhesion and other essential biological processes (Fig. 6b). In addition, Cytoscape was used to determine the relationship for the enriched terms and to build a network diagram (Fig. 6c and d).

## Discussion

ITGA5 belongs to the integrin alpha chain family and interacts with ITGB1 to form a heterodimeric integral membrane protein - integrin α5β1 complex that performs diverse biological functions. Despite not knowing all of the functions performed by ITGA5, multiple studies have demonstrated that increased expression of ITGA5 is associated with poor prognosis in multiple tumors types, such as triple negative breast cancer [14], lung cancer [35], hepatocellular carcinoma [36] and ovarian cancer [37]. Here, public databases were used to determine ITGA5 expression levels in different cancer types. Compared to normal tissues, ITGA5 was generally overexpressed in different cancers including multiple types of gastrointestinal tumors. We explored whether the expression of ITGA5 was correlated with patient prognosis in these different tumors. Results revealed that high expression levels of ITGA5 were significantly correlated with worse OS and DFS in four types of gastrointestinal tumors, including colorectal, pancreatic, gastric and liver cancers. Although the results did not show statistical significance, esophageal cancer patients with high ITGA5 expression levels still showed a trend of high survival risk. To understand the clinical significance of ITGA5 in gastric cancer, the prognostic values for ITGA5 expression levels with various clinicopathological factors were determined. High expression of ITGA5 was correlated with poor prognosis in gastric cancer stages II-IV, T2-T4, N1-N3 and M0-M1. These results demonstrated that ITGA5 has potential as a prognostic biomarker in gastrointestinal tumors.

Both innate and adaptive immune cells regulate the biological behavior of tumors and the response to treatment through direct contact or signal transduction mechanisms [17]. We therefore explored the relationship between ITGA5 expression levels and immune infiltration levels. Our results indicated that ITGA5 expression was significantly and negatively correlated with immune purity in four types of gastrointestinal tumors, including COAD, ECAD, STAD and READ. In STAD and COAD, significant and positive relationships were observed between ITGA5 expression and infiltration levels for CD4 + T cells, macrophages, neutrophils and dendritic cells. In addition, the relationship between ITGA5 expression levels and immune cell markers implicates underlying regulation in STAD and COAD. First, gene markers for Th2 cells (GATA3, STAT6, STAT5A and IL13) showed a positive correlation with ITGA5 expression. The M2 macrophage markers (CD163, VSIG4 and MS4A4A) showed a moderate or strong correlation. In addition, we also observed a significant correlation between ITGA expression and monocytes and TAMs in COAD and STAD. These results revealed a potential regulatory role for ITGA5 in the polarization of macrophages. TAMs, derived from mononuclear cells, are induced to differentiate into M2 cells by cytokines such as IL-4, IL-10 and IL-13, which are secreted by Th2 cells [38–40]. Moreover, M2 cells exhibit protumoral activity by promoting genetic instability, angiogenesis, stem cell nurturing and local immunosuppression [41, 42]. Subsequently, IHC and western blot were performed on adjacent normal and tumor tissues extracted from gastric cancer patients to find that Th2 cell markers such as STAT6, GATA3 and the M2 cell polarization marker CD163 were significantly increased in patients with high ITGA5 expression levels. This suggested that ITGA5 may play an important role in promoting the recruitment and activation of monocytes, TAMs, Th2 cells and M2 cells in the tumor microenvironment.

Emerging evidence provides potential mechanisms to explain the correlation between ITGA5 expression levels and immune infiltration. Integrin α5β1 usually acts as a receptor for fibronectin to perform biological functions. Previous studies mainly focused on the role of ITGA5 in tumor cells, but recent studies found that ITGA5 is also expressed in cancer-associated fibroblasts [43], TAMs [44] and chimeric antigen receptor expressing T cells [45]. Based on previous research, cancer-associated fibroblasts, which are regulators of immune cell recruitment and function, affect both innate and adaptive immune responses [46, 47]. Similarly, ITGA5 is expressed on the surface of TAMs and chimeric antigen receptor expressing T cells and can directly regulate the recruitment and alternative activation of immune cells. Interestingly, further work has shown that decreased expression of ITGA5 down-regulates pancreatic stellate cells differentiation into cancer-associated fibroblasts in pancreatic ductal cancer [15]. Importantly, pancreatic stellate cell are seen as the main source of cancer-associated fibroblasts [48]. The study presented here revealed an important role for ITGA5 in the differentiation and maturation of cells. These results partly explain the effects of ITGA5 expression on immune cell infiltration in malignant tumors and provides insight for further work. ITGA5 and its interacting proteins play an important role in integrin-mediated signaling pathways, leukocyte migration and cell-matrix adhesion as shown by the PPI network that was constructed as well as gene functional enrichment analysis. ITGA5 may act as an “anchor” for cell positioning, promoting the aggregation, adhesion and migration of partial immune cells and changing components of the tumor microenvironment. This may result in immune escape and suppression, ultimately accelerating tumor development and metastasis. In the future, the possible role and mechanisms behind ITAG5 in tumor immunity should be explored.

## Conclusions

In summary, our study demonstrated that ITGA5 may be an essential regulator of tumor immune cell infiltration and a valuable prognostic biomarker in gastrointestinal tumors. Additional work is needed to fully elucidate the underlying mechanisms behind these observations.

## Supplementary Information

**Additional file 1 **: **Table S1**. The information of antibody for immunohistochemistry. **Table S2**. The information of antibody for western blot.

**Additional file 2 **: **Figure S1**. The prognostic value of ITGA5 expression in non-gastrointestinal cancers. (A-D) Correlation between ITGA5 expression and prognosis of lung cancer and breast cancer in Kaplan-Meier Plotter; (E-F) Correlation between ITGA5 expression and prognosis of ovarian cancer in PrognoScan. OS, overall survival; DFS, disease free survival. **Figure S2**. The full-length, original blots for CD163 (A), ITGA5 (B), GATA3 (C) and STAT6 (D) in 6 paired gastric cancer tissues (T) and adjacent normal tissues (N). (the order of samples from left to right are: marker, N1, T1, N2, T2, N3, T3, marker, N4, T4, N5, T5, N6, T6, marker).

## Data Availability

The datasets used and/or analyzed during the current study are available from the corresponding author on reasonable request.

## Abbreviations

ITGA5: Integrin subunit alpha 5
TAMs: Tumor - associated macrophages
ITGB1: Integrin subunit beta 1
TME: Tumor microenvironment
PPI: Protein-protein interaction
GO: Gene ontology
KEGG: Kyoto encyclopedia of genes and genomes
OS: Overall survival
DFS: Disease free survival
PFS: Progression free survival
HR: Hazard ratio
CI: Confidence interval
TCGA: The cancer genome atlas
NK: Natural killer
DCs: Dendritic cells
Th1: T-helper 1
Th2: T-helper 2
Tfh: Follicular helper T
Th17: T-helper 17
GEPIA2: Gene expression profiling interactive analysis 2
IHC: Immunohistochemistry
LIHC: Liver hepatocellular carcinoma
STAD: Stomach adenocarcinoma
COAD: Colon adenocarcinoma
ESCA: Esophageal carcinoma
READ: Rectum adenocarcinoma
PAAD: Pancreatic adenocarcinoma
