# Supplementary material for: ITGA5 is a prognostic biomarker and correlated with immune infiltration in gastrointestinal tumors
Source: BMC Cancer. 2021 Mar 12;21:269. doi: 10.1186/s12885-021-07996-1 (PMC7953822; doi:10.1186/s12885-021-07996-1)
Supplement: Supplementary file 1 — Additional file 1 : Table S1. The information of antibody for immunohistochemistry. Table S2. The information of antibody for western blot. [file 12885_2021_7996_MOESM1_ESM.docx]

| **Additional file 1: Table S1** \| The information of antibody for immunohistochemistry | | | |
| --- | --- | --- | --- |
| **Antibody** | **Company** | **Catalog** | **Concentration** |
| **ITGA5** | Proteintech | 27224-1-AP | 1:500 |
| **CD163** | Proteintech | 16646-1-AP | 1:2000 |
| **STAT6** | Proteintech | 51073-1-AP | 1:100 |
| **GATA3** | Proteintech | 10417-1-AP | 1:200 |

| **Additional file 1: Table S2**\| The information of antibody for western blot | | | |
| --- | --- | --- | --- |
| **Antibody** | **Company** | **Catalog** | **Concentration** |
| **ITGA5** | Abcam | ab150361 | 1:2000 |
| **CD163** | Abcam | ab87099 | 1:1000 |
| **STAT6** | Abcam | ab32520 | 1:1000 |
| **GATA3** | Cell Signal Technology | 5852 | 1:1000 |
| **GAPDH** | Proteintech | 10494-AP | 1:10000 |
