# Supplementary material for: ITGA5 is a prognostic biomarker and correlated with immune infiltration in gastrointestinal tumors
Source: BMC Cancer. 2021 Mar 12;21:269. doi: 10.1186/s12885-021-07996-1 (PMC7953822; doi:10.1186/s12885-021-07996-1)
Supplement: Supplementary file 2 — Additional file 2 : Figure S1. The prognostic value of ITGA5 expression in non-gastrointestinal cancers. (A-D) Correlation between ITGA5 expression and prognosis of lung cancer and breast cancer in Kaplan-Meier Plotter; (E-F) Correlation between ITGA5 expression and prognosis of ovarian cancer in PrognoScan. OS, overall survival; DFS, disease free survival. Figure S2. The full-length, original blots for CD163 (A), ITGA5 (B), GATA3 (C) and STAT6 (D) in 6 paired gastric cancer tissues (T) and adjacent normal tissues (N). (the order of samples from left to right are: marker, N1, T1, N2, T2, N3, T3, marker, N4, T4, N5, T5, N6, T6, marker). [file 12885_2021_7996_MOESM2_ESM.zip › Additional file Figure S1.pdf]

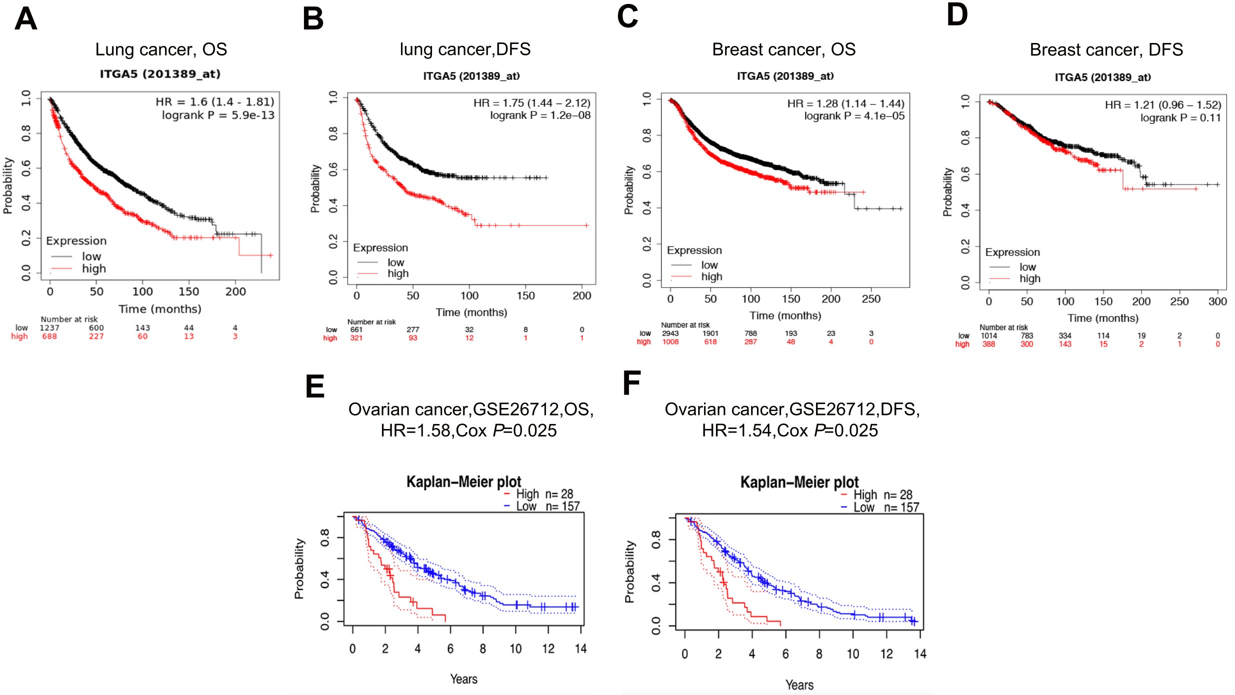

A
Lung cancer, OS
ITGA5 (201389_at)
HR = 1.6 (1.4 - 1.81)
logrank P = 5.9e-13
Expression
low
high
Probability
Time (months)
Number at risk
low 1237 600 143 44 4
high 688 227 60 13 3
B
lung cancer,DFS
ITGA5 (201389_at)
HR = 1.75 (1.44 – 2.12)
logrank P = 1.2e–08
Expression
low
high
Probability
Time (months)
Number at risk
low 661 277 32 8 0
high 321 93 12 1 1
C
Breast cancer, OS
ITGA5 (201389_at)
HR = 1.28 (1.14 – 1.44)
logrank P = 4.1e–05
Expression
low
high
Probability
Time (months)
Number at risk
low 2943 1901 788 193 23 3
high 1008 618 287 48 4 0
D
Breast cancer, DFS
ITGA5 (201389_at)
HR = 1.21 (0.96 – 1.52)
logrank P = 0.11
Expression
low
high
Probability
Time (months)
Number at risk
low 1014 783 334 114 19 2 0
high 388 300 143 15 2 1 0
E
Ovarian cancer,GSE26712,OS,
HR=1.58,Cox P=0.025
Kaplan–Meier plot
High n= 28
Low n= 157
Probability
Years
F
Ovarian cancer,GSE26712,DFS,
HR=1.54,Cox P=0.025
Kaplan–Meier plot
High n= 28
Low n= 157
Probability
Years
