# Supplementary figures and images for: ITGA5 is a prognostic biomarker and correlated with immune infiltration in gastrointestinal tumors
Source: BMC Cancer. 2021 Mar 12;21:269. doi: 10.1186/s12885-021-07996-1 (PMC7953822; doi:10.1186/s12885-021-07996-1)

**A**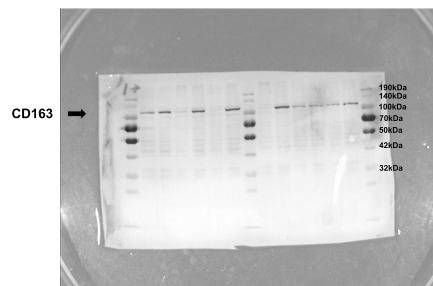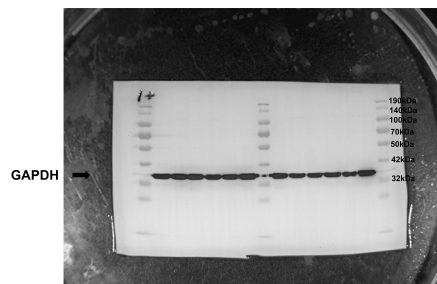**B**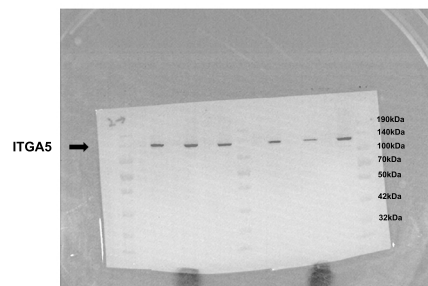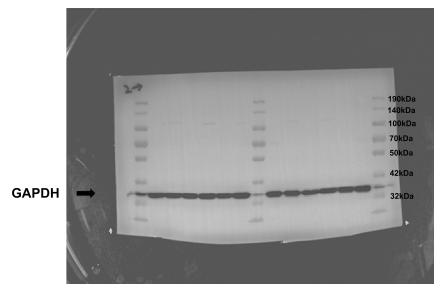**C**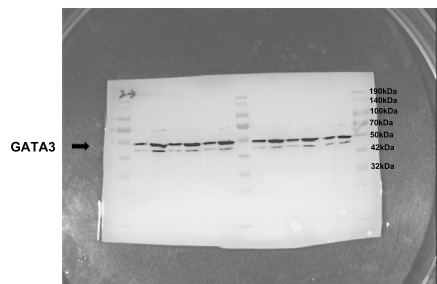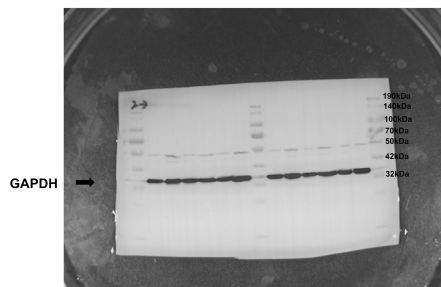**D**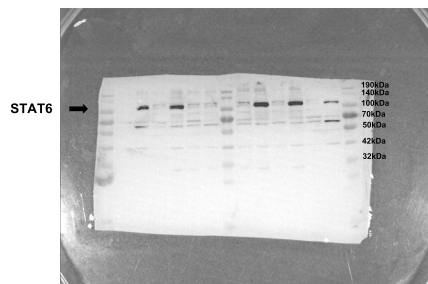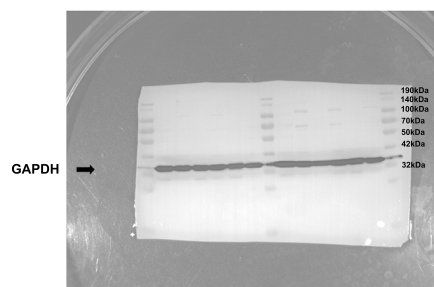

Supplement: Supplementary file 2 — Additional file 2 : Figure S1. The prognostic value of ITGA5 expression in non-gastrointestinal cancers. (A-D) Correlation between ITGA5 expression and prognosis of lung cancer and breast cancer in Kaplan-Meier Plotter; (E-F) Correlation between ITGA5 expression and prognosis of ovarian cancer in PrognoScan. OS, overall survival; DFS, disease free survival. Figure S2. The full-length, original blots for CD163 (A), ITGA5 (B), GATA3 (C) and STAT6 (D) in 6 paired gastric cancer tissues (T) and adjacent normal tissues (N). (the order of samples from left to right are: marker, N1, T1, N2, T2, N3, T3, marker, N4, T4, N5, T5, N6, T6, marker). [file 12885_2021_7996_MOESM2_ESM.zip › Additional file Figure S2.pdf]
